# Genome-Wide Distribution and Organization of Microsatellites in Plants: An Insight into Marker Development in *Brachypodium*

**DOI:** 10.1371/journal.pone.0021298

**Published:** 2011-06-21

**Authors:** Humira Sonah, Rupesh K. Deshmukh, Anshul Sharma, Vinay P. Singh, Deepak K. Gupta, Raju N. Gacche, Jai C. Rana, Nagendra K. Singh, Tilak R. Sharma

**Affiliations:** 1 National Research Centre on Plant Biotechnology, Indian Agricultural Research Institute (IARI), New Delhi, India; 2 National Bureau of Plant Genetic Resources, Regional Station, Shimla (HP), India; 3 Swami Ramanand Teerth Marathwada University, Nanded, Maharashtra, India; University of Georgia, United States of America

## Abstract

Plant genomes are complex and contain large amounts of repetitive DNA including microsatellites that are distributed across entire genomes. Whole genome sequences of several monocot and dicot plants that are available in the public domain provide an opportunity to study the origin, distribution and evolution of microsatellites, and also facilitate the development of new molecular markers. In the present investigation, a genome-wide analysis of microsatellite distribution in monocots (*Brachypodium*, sorghum and rice) and dicots (*Arabidopsis*, *Medicago* and *Populus*) was performed. A total of 797,863 simple sequence repeats (SSRs) were identified in the whole genome sequences of six plant species. Characterization of these SSRs revealed that mono-nucleotide repeats were the most abundant repeats, and that the frequency of repeats decreased with increase in motif length both in monocots and dicots. However, the frequency of SSRs was higher in dicots than in monocots both for nuclear and chloroplast genomes. Interestingly, GC-rich repeats were the dominant repeats only in monocots, with the majority of them being present in the coding region. These coding GC-rich repeats were found to be involved in different biological processes, predominantly binding activities. In addition, a set of 22,879 SSR markers that were validated by e-PCR were developed and mapped on different chromosomes in *Brachypodium* for the first time, with a frequency of 101 SSR markers per Mb. Experimental validation of 55 markers showed successful amplification of 80% SSR markers in 16 *Brachypodium* accessions. An online database ‘BraMi’ (*Brachypodium* microsatellite markers) of these genome-wide SSR markers was developed and made available in the public domain. The observed differential patterns of SSR marker distribution would be useful for studying microsatellite evolution in a monocot–dicot system. SSR markers developed in this study would be helpful for genomic studies in *Brachypodium* and related grass species, especially for the map based cloning of the candidate gene(s).

## Introduction

Microsatellites or simple sequence repeats (SSRs) are co-dominant, abundant, multi-allelic, and uniformly distributed over the genome, and can be detected by simple reproducible assays [1]. These important features have made microsatellites the markers of choice for marker-assisted plant breeding, DNA fingerprinting of genetic resources, molecular mapping and map based cloning of specific genes. Microsatellite markers have also been used in several studies to define conserved regions among related species [2]–[4]. Initially, SSR markers were developed from expressed sequence tags (ESTs) and bacterial artificial chromosome (BAC) end sequences in most plant species. However, whole genome sequencing has led to the identification of numerous SSR markers that are distributed over the entire genomes of rice and *Arabidopsis*. The mechanism of microsatellite evolution and their genome-wide distribution, however, are still not well studied in plants mostly due to the lack of genomic information. The recently sequenced genomes of *Brachypodium* [5], *Populus* [6], *Medicago* (www.jgi.doe.gov) and sorghum [7] along with the already well-characterized sequenced genomic information available for rice [8] and *Arabidopsis* [9], will facilitate the comparative genomics studies in plants. Besides understanding genome organization, sequenced genomes can be effectively used for the generation of molecular markers and their cross species utilization, specifically for those species where very little or no genomic information is available.

Thousands of SSR markers have been developed in rice and their application in other grass species has been proven and well documented [10]-[12]. However, it is postulated that the *Brachypodium* genome would exhibit a higher level of collinearity to the genomes of temperate cereal crops as compared to the rice genome. Therefore, SSR markers developed in *Brachypodium* may be utilized more effectively in wheat compared to rice SSR markers. Moreover, several features such as a typical plant structure, growth habit, rapid generation time, self pollination and a compact genome size (∼300 Mb) make *Brachypodium* an excellent model system for functional and structural genomic studies in cereals and grasses [13]–[14]. Several efforts have been made to make *Brachypodium* a more useful and convenient model system for genomic studies [14]. However, to develop any model system for genomic studies, a dense molecular genetic linkage map and genome-wide distributed molecular markers are required. Recently, a genetic linkage map of 139 marker loci for *Brachypodium* was developed using F_2_ population derived from a cross between diploid lines Bd3-1 and Bd21 [15]. The map was constructed using SSR markers derived from the EST, BAC end sequences and information available on conserved orthologous sequence (COS) in other grass species. Currently, efforts are underway to develop a dense molecular genetic linkage map for *Brachypodium* using different types of markers. However, a genome-wide resource of a large number of informative molecular markers is required to supplement this effort.

The present study deals with a genome-wide comparative analysis of microsatellite distribution in the nuclear and chloroplast genomes of monocot and dicot species, the development of genome-wide SSR markers, and the validation of a subset of these new markers in *Brachypodium*.

## Results and Discussion

### Microsatellite distribution in monocot and dicot species

A total of 797,863 SSRs were identified among six plant genomes — three monocot (*Brachypodium*, sorghum and rice) and three dicot (*Arabidopsis*, *Medicago* and *Populus*) plant species (Table 1). Among the six genomes analyzed, the maximum number and frequency of SSRs were obtained from *Populus* followed by *Medicago* whereas the sorghum genome had the lowest frequency. The frequency of SSRs was considerably higher among dicots compared to monocots. Among monocots, the frequency of SSRs in the rice genome was nearly twice that in sorghum and *Brachypodium* genomes (Table 1). Since the six selected plant species belonged to very diverse groups of monocots and dicots, the distribution pattern of SSR motifs, with specific sequences in these genomes, was not uniform. However, the overall pattern of SSR motifs of particular lengths was similar. Mono-nucleotide repeats dominated over other type of repeats in all the six plant species. However, the frequency of SSRs decreased stepwise with increase in motif length (mono- to hexa- nucleotide repeats) except in *Brachypodium* where the frequency of tri- nucleotide repeats was higher than that of the di-nucleotide repeats (Figure S1). Mono-nucleotide repeats were found to be minimum (43%) in sorghum and maximum (79%) in *Medicago* genomes. While the mono-, di- and tri-nucleotide repeats mostly contributed to the major proportion of SSRs, a very small share was contributed by tetra-, penta- and hexa-nucleotide repeats. A maximum of 5.4% contribution of tetra-, penta- and hexa- nucleotide repeats, was observed in the sorghum genome. A similar trend was observed for other genomes studied in the present investigation (Table 1).

**Table 1:** Distribution of microsatellite with respect to motif length and genome size in monocot and dicot plant species.

Plant Species	Size analyzed (bp)	Number of motif repeats						Total	SSR/Mb	
		Mono	Di	Tri	Tetra	Penta	Hexa			
***B. distachyon***	271,148,426*	30,573	9,407	10,625	990	196	84	51,875		191.3
***S. bicolor***	738,540,932*	55,906	38,138	28,480	5,368	946	726	1,29,564		175.4
***O. sativa***	372,317,567#	64,734	37,282	29,819	2,565	604	261	1,35,265		363.3
***A. thaliana***	119,667,751$	34,843	9,386	5,596	169	41	57	50,092		418.6
***M. truncatula***	307,481,907*	1,20,383	20,999	9,647	1,079	216	137	1,52,461		495.8
***P. trichocarpa***	417,137,944*	194,557	54,304	25,130	3,178	772	665	2,78,606		667.9
**Total**	**2,226,294,527**	**500,996**	**169,516**	**109,297**	**13,349**	**2,775**	**1,930**	**7,97,863**	**358.38**	

*www.phytozome.net

# www.rice.plantbiology.msu.edu

$ www.arabidopsis.org

Among the two types of mono-nucleotide repeats, (A/T)n was the most abundant in all the plant species while (G/C)n was comparatively scarce (Table 2). In the mono-nucleotide repeats category, the maximum (99%) A/T repeats were present in the *Arabidopsis* genome and the minimum (78%) in the *Brachypodium* genome. In the di-nucleotide repeat category, the distribution of SSRs in different motif types was not uniform and the most frequent motif type was different for each plant species. For example, AG/CT repeats were more frequent in *Brachypodium* and rice with 50.7% and 41.9% frequency, respectively; whereas AT/AT repeats were more frequent in *Populus* (60.5%) and *Medicago* (59.9%). In rice, both AG/CT and AT/AT repeats dominated other di-nucleotide repeats. Interestingly, the CG/CG motif contributed less than 0.5% in dicots, whereas it was 3.1%–7.0% in all di-nucleotide repeats identified in the monocots. The analysis of mono- and di-nucleotide repeats concluded that CG-rich motifs were least preferred in both monocot and dicot genomes. However, for tri-nucleotide repeats the AGC/CGT, AGG/CCT and CCG/CGG were observed more frequently in all the monocot species, whereas A/T-rich repeats, such as AAC/GTT, AAG/CTT and AAT/ATT, were preferred in dicots (Table 2). The frequency of tetra-, penta- and hexa-nucleotide repeats was very low in all the plant genomes investigated in the present study and their motif-wise distribution was not significant across the genomes.

**Table 2:** Frequency of different types of motifs in a class of microsatellites with mono-, di-, and tri-nucleotide repeats analysed in monocot and dicot plant genomes.

Repeat type	Monocot			Dicot	
	*B. distachyon*	*S. bicolor*	*O.* *sativa*	*A. thaliana*	*M. truncatula*	*P.* *trichocarpa*
**Mono-nucleotide**						
A/T	78.0	85.7	86.3	99.0	98.5	98.02.0
C/G	22.0	14.3	13.7	1.0	1.5	
**Di-nucleotide**						
AC/GT	21.7	14.0	10.0	10.6	13.0	14.9
AG/CT	50.7	28.5	41.9	36.8	26.8	24.1
AT/AT	22.4	54.4	41.0	52.6	59.9	60.5
CG/CG	5.2	3.1	7.0	0.0	0.3	0.5
**Tri-nucleotide**						
AAC/GTT	5.4	7.5	1.7	13.1	18.6	6.5
AAG/CTT	16.7	11.2	6.2	46.8	21.3	21.6
AAT/ATT	3.2	12.7	4.5	7.9	38.5	48.2
ACC/GGT	3.9	5.0	6.0	4.3	4.5	3.9
ACG/CTG	9.2	11.1	8.4	1.8	1.1	2.3
ACT/ATG	2.3	5.7	2.2	8.9	5.7	4.1
AGC/CGT	9.2	11.6	8.4	1.7	1.1	2.3
AGG/CCT	15.8	11.3	13.2	6.3	2.9	6.5
AGT/ATC	2.2	5.9	2.0	8.6	6.0	4.2
CCG/CGG	32.1	18.1	47.5	0.6	0.2	0.6

Data are percentage of SSR in particular class.

The dominant occurrence of repeat motifs, of a particular sequence and length, in plant genomes is the outcome of selection pressures applied on that specific motif during evolution. The molecular mechanisms for the origin of microsatellites are not completely understood. The most common mutational mechanism affecting microsatellites is replication slippage, a process involving addition or removal of one or more motif repeats; however other mechanisms, such as unequal crossing over, nucleotide substitutions, or duplication events, have also been considered to be responsible for microsatellite variations [16]–[18]. However, these theories cannot explain the species-specific accumulation of particular motif repeats observed in the present study. Other factors, such as codon preference, DNA replication and the mismatch repair system, as well as structural and functional attributes of genomes that are unique to the species or for the particular taxon, may be responsible for the unique microsatellite distribution patterns in plant genomes. Moreover, the SSR length, motif structure and G/C content of a genome are considered to be factors influencing microsatellite evolution [19]–[21]. Polymorphism among SSRs is a repeat length polymorphism due to repeat elongation/shortening events, which indicates that such processes are important factors for molecular evolution. The repeat elongation/shortening processes also lead to increase in biological complexity, which is a characteristic of biological evolution. It is known that SSRs within genes are substantially involved in the regulation of evolutionary processes as SSRs in the protein-coding regions can lead to a gain or loss of gene function. Earlier, sequence variations in genomes, particularly in microsatellite distribution, were supported by the theory of stabilization patterns and potential secondary structures, as well as factors such as the mismatch repair enzymes [22]–[24]. All these theories, which deal with a particular factor being responsible for sequence preference, were suggested based on very limited knowledge and lack of genome-wide information on a large variety of genomes. Till date, very little work has been done to propose a genome-wide mechanism for the selection of microsatellite motifs with a particular sequence. These mechanisms may be further illustrated with available genomic resources, and the data presented in this paper would definitely help in the understanding of microsatellite evolution in the genomes of plant species.

### Microsatellite distribution in coding regions

**Figure 1:**
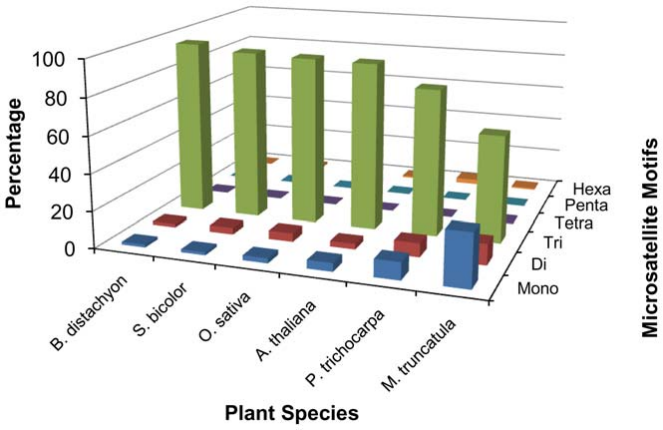
Distribution of microsatellites in coding DNA sequences (CDS) of six plant species with respect to motif length. Microsatellites were identified with criteria of mono- to hexa-nucleotides motifs using MISA software tool, and the minimum repeat unit was defined as 10 for mono-, 6 for di-, and 5 for tri-, tetra-, penta-, and hexa-nucleotides.

Microsatellites were identified in the coding DNA sequences (CDS) of six plant species to study the pattern of distribution in the coding regions of monocots and dicots. A total of 36,585 SSRs were identified in the 238,798 CDS of about 269.3 Mb size data for all the six plant species included in this study. Interestingly, the frequency of SSRs observed in the CDS region (CDS-SSRs) of monocots was twice that observed in the CDS of dicots (Table S1). The highest frequency (203.7 SSR/Mb) of CDS-SSRs was identified in rice followed by sorghum (181.1 SSR/Mb) whereas the lowest frequency (68.1 SSR/Mb) was observed in *Populus*. Tri-nucleotide repeats were found to be most abundant among the microsatellites in the coding region of plant genomes (Figure 1) and contributed to about 93% of SSRs in monocots and about 76% of SSRs in dicots. Such an accumulation of tri-nucleotide repeats in the coding regions was mostly due to the triplet-repeat nature of the codon. Mono-nucleotide repeats contributed about 2% of such SSRs in monocots while in dicots it was 14.2%; this variation may be due to the more frequent occurrence of A/T repeats in dicots. Moreover, G/C-rich repeats in the CDS region of monocots were identified with much more frequency than in dicots (Table 3). In the category of mono-nucleotide repeats, A/T repeats dominated over G/C repeats in both monocots and dicots except in rice where G/C repeats contributed to 53.1% of mono-repeat SSRs. Although in monocots G/C repeats were slightly less than A/T repeats, these were very infrequent in dicots, and as low as 3.4% in *Arabidopsis* (Table 3). Interestingly, in the category of di-nucleotide repeats, GC/CG repeats were predominant in monocots with an average of 49.9%, while they were completely absent in dicots. The AG/CG repeats accounted for an average of 41.1% SSRs in monocots and were also major contributors in dicots with an average of 73.5% di-nucleotide SSRs. Tri-nucleotides with repeat motifs CCG/GGC were dominant only in monocots and contributed to 51.5% of the total tri-nucleotide repeats identified in monocots, whereas only 1.9% of these repeats were present in dicots. Tri-nucleotides with repeat motifs AAG/CTT accounted for 29.5% of the total tri-nucleotide repeats in dicots. The microsatellite distribution pattern in the CDS region was found to be very unique for the monocot and dicot species. Functional annotation of *Brachypodium* CDS sequences containing CG-rich repeats revealed that about 50% of these genes were involved in binding activity (Table S2).

**Table 3:** Frequency of microsatellite motifs in coding DNA sequence of six plant species.

Repeat type	Monocot			Dicot	
	*B. distachyon*	*S. bicolor*	*O.* *sativa*	*A. thaliana*	*M. truncatula*	*P. trichocarpa*
**Mono-nucleotide**						
A/T	35	65	180	114	848	252
C/G	23	44	204	4	122	27
**Di-nucleotide**						
AC/GT	5	39	13	7	55	22
AG/CT	18	79	313	61	273	170
AT/AT	1	23	8	11	57	30
CG/CG	51	106	341	0	0	0
**Tri-nucleotide**						
AAC/GTT	94	64	128	297	280	134
AAG/CTT	244	227	392	885	565	419
AAT/ATT	3	12	3	11	72	49
ACC/GGT	144	424	878	187	278	411
ACG/CTG	275	752	1,037	53	76	174
ACT/ATG	72	100	234	325	274	241
AGC/CGT	548	1004	1,361	0	83	66
AGG/CCT	546	845	1,718	302	170	405
AGT/ATC	19	26	47	201	177	90
CCG/CGG	1,697	2,782	7,433	27	14	80

Monocots and dicots are thought to have diverged from a common ancestor approximately 200 million years ago [25]. In several comparative genomic studies, *Arabidopsis* and rice have been considered as models for dicots and monocots, respectively. However, numerous interesting findings have emerged while comparing these two genomes; for example, rice genes are longer and GC-rich than *Arabidopsis* genes [26]. Though *Arabidopsis* has the smallest genome among the dicot species, it is thought to have evolved by chromosomal duplication; while the rice genome, which is comparatively larger than the *Arabidopsis* genome, showed more duplication [26]–[29]. The GC-rich monocot genomes may have microsatellites with GC-rich motifs whereas dicots lack GC-rich motifs. The relationship between microsatellite evolution and chromosomal duplications has not been well studied. The duplicated regions are thought to have different selection pressures than other regions, which may be a reason for motif preference and frequency in monocots and dicots. Such a biased selection of SSRs was observed in *Populus* where most of the SSRs in the coding regions are missing at the duplicated chromosomal segment mostly due to loss of corresponding genes [30]. This emphasizes the role of microsatellites in gene and genome evolution. Although, a systematic study on a number of genomes is required to make any definite conclusions, recent developments in sequencing technology and the availability of an increasing number of genome sequences for analysis would definitely provide a basis for the study of microsatellite evolution in plants [31].

### SSRs frequency in plant chloroplast genomes

**Figure 2:**
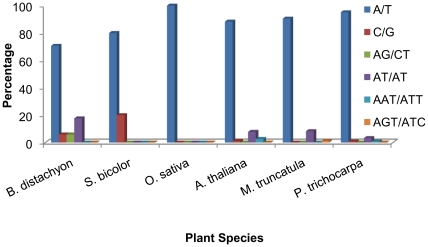
Distribution of microsatellites in the chloroplast genome of six plant species with respect to motif type. Microsatellites were identified with criteria of mono- to hexa-nucleotides motifs using MISA software tool, and the minimum repeat unit was defined as 10 for mono-, 6 for di-, and 5 for tri-, tetra-, penta-, and hexa-nucleotides.

A total of 337 SSRs were identified for the chloroplast genome of the six plant species analyzed in this study. The highest frequency of SSRs was identified in the chloroplast genome of *Populus* followed by *Medicago* and *Arabidopsis* (Table S3). Compared with dicots, monocots had very infrequent SSRs in the chloroplast genome. Most of the SSRs identified in the plant chloroplast genome were mono-nucleotide repeats that contributed about 92.5% of the total SSRs (Figure 2). In the mono-nucleotide category of repeats, A/T contributed 97.4% of the total repeats. For nuclear genomes, G/C repeats were predominantly found in the sorghum and *Brachypodium* chloroplast genomes. Although, the number of di- and tri-nucleotide repeats identified in the chloroplast genome is not enough to compare patterns in the chloroplasts of monocots and dicots, in a broader context the chloroplasts of dicots were richer in SSRs with di- and tri-nucleotide repeats, which were otherwise lacking in monocots.

### SSRs frequency in *Brachypodium* genomes

A total of 51,875 SSRs were identified in the 271 Mb sequence of the *Brachypodium* genome with a maximum of 30,573 mono-, followed by 10,625 di- and 9,407 tri-nucleotide repeats (Table 4). The penta- and hexa-nucleotide repeats were only 1,270, which represented 2.5% of the total SSRs identified in the *Brachypodium* genome. Chromosome 4 of *Brachypodium* contained the maximum frequency (197 SSRs/Mb) of SSRs, whereas chromosome 5 contained the minimum frequency (175 SSRs/Mb) of SSRs. The frequency of SSRs in the different chromosomes was almost uniform and the overall frequency of SSRs in *Brachypodium* was 191 SSRs/Mb.

**Table 4:** Chromosome-wide distribution of microsatellites in the *B. distachyon* genome.

SSR type	Chromosome					Total(No)
	1	2	3	4	5	
**Mono** 1	8,529	6,746	6,710	5,552	3,036	30,573
**Di** 1	2,663	2,037	2,050	1,762	895	9,407
**Tri** 1	2,945	2,306	2,394	2,042	938	10,625
**Tetra** 1	280	218	228	169	95	990
**Penta** 1	45	39	45	47	20	196
**hexa** 1	26	18	12	20	8	84
**Size (Mb)**	74.83	59.33	59.89	48.65	28.44	271.15
**SSR/Mb**	193.6	191.5	191.0	197.2	175.5	191.3
**Total SSR**	**14,488**	**11,364**	**11,439**	**9,592**	**4,992**	**51,875**

1 **-nucleotides.**

Interestingly, the frequency of SSRs observed on the short arm of chromosome 5 was much lower than that on the long arm. This low frequency of SSRs in the short arm was common for all types of motifs (Figure S2). The short arm of chromosome 5 (Bd5s) has several features that are different from the rest of the chromosomes [5]. These include a low gene density (roughly half of the rest of the chromosomes); a high LTR retrotransposon density with the youngest intact Gypsy elements; and the lowest solo LTR density. These attributes may be responsible for the low frequency of microsatellites in Bd5s.

### Development of genome-wide SSR markers and their validation in *Brachypodium* genotypes

**Figure 3:**
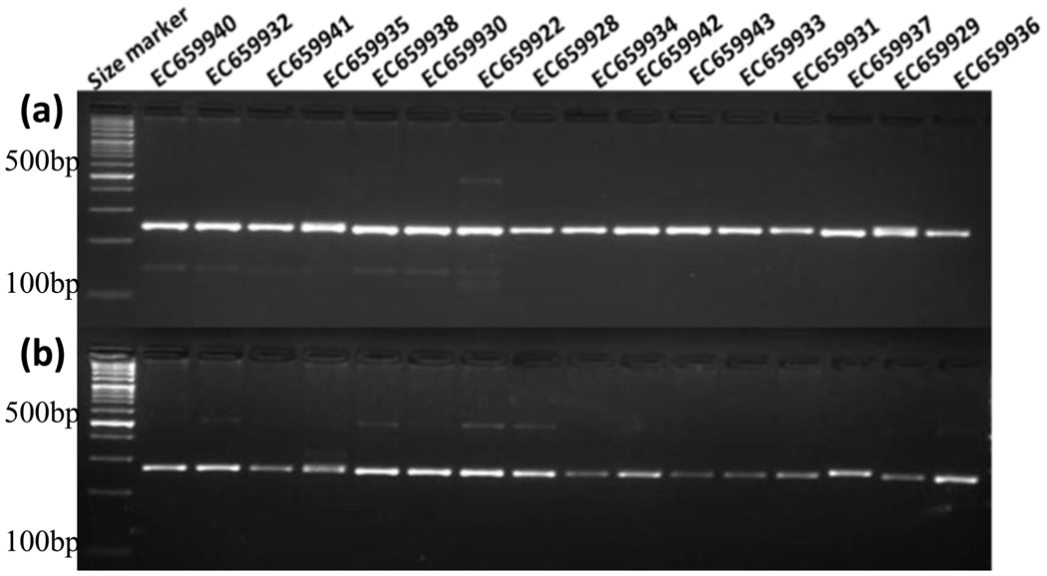
PCR-amplified product of marker (a) B2M569 and (b) B2M1 for the 16 Brachypodium genotypes resolved on the 3% metaphor agarose gel.

A total of 27,329 SSR markers (including 7,225 class I and 20,104 class II) were successfully designed. Of these 22,879 (83.7%) SSR markers were validated by e-PCR (Table 5, Table S4). A subset of 44 (80%) markers was amplified in 16 *Brachypodium* genotypes with prominent PCR products of expected size (Figure 3, Table S5).

**Table 5:** Summary of the primers designed for microsatellites of different motif types in *B. distachyon* genome.

SSR type /motif	No. of markers in each chromosome					Total(No)	e-PCRvalidated
	1	2	3	4	5		
**Class I**							
**Mono**	174	135	131	108	71	619	569
**Di**	379	276	278	245	110	1,288	1,076
**Tri**	374	306	315	267	149	1,411	1,167
**Tetra**	244	193	196	143	78	854	821
**Penta**	39	34	44	40	17	174	134
**Hexa**	20	14	10	18	7	69	55
**Compound**	798	642	600	510	260	2,810	2,278
**Total**	**2,028**	**1,600**	**1,574**	**1,331**	**692**	**7,225**	**6,100**
**Class II (all)**	5,617	4,376	4,448	3,798	1,865	20,104	16,779
**Grand Total**	**7,645**	**5,976**	**6,022**	**5,129**	**2,557**	**27,329**	**22,879**

**Figure 4:**
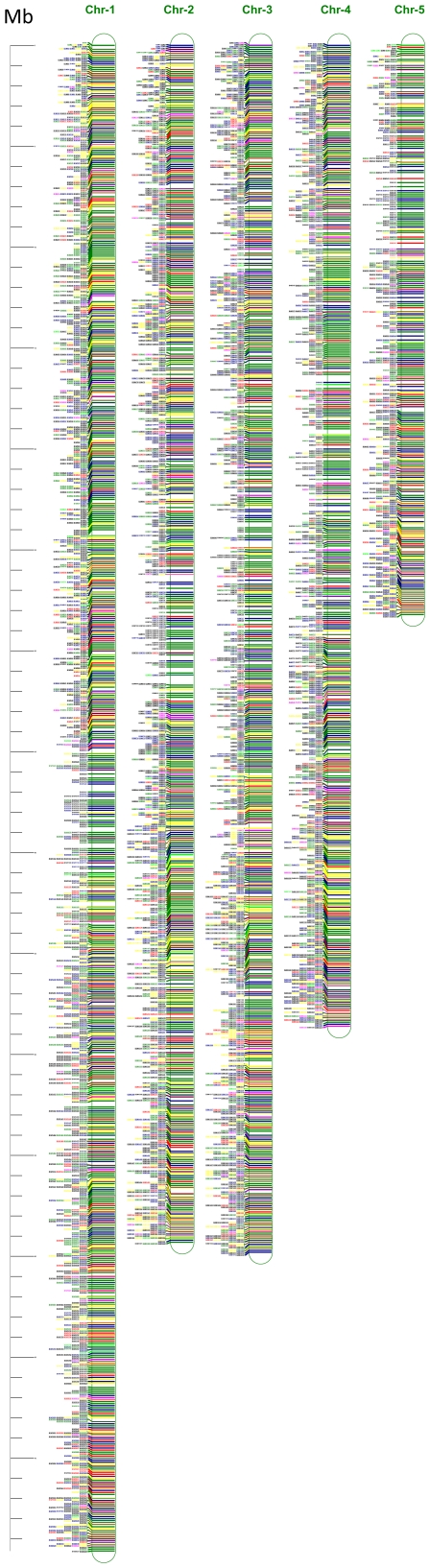
Distribution of SSR markers developed for the five chromosomes of *Brachypodium distachyon* genome. Markers were presented on the left hand side of the chromosome bar as per the physical location and named (e.g. B01d12) using prefix B for *Brachypodium* followed by chromosome number then d for *distachyon*, followed by the number of SSRs as per their order on the chromosome. The left vertical bar is the physical length ruler scaling in Mb.

The frequency of SSR markers was 101 per Mb, covering the entire genome with very fine gaps of less than 10 Kb (Figure 4). Most of these gaps were found at the centromeric regions. The primer set also included 2,810 compound SSRs, which would provide better polymorphism in *Brachypodium* (Table 5). The rationale for categorizing SSRs as Class I and Class II was that the SSRs with the larger number of repeats (Class I) were found to be more polymorphic than those with lesser number of repeats (Class II) in animals [32]–[33] as well as in plants [34]. These results from animals and plants were concluded on the basis of polymorphism observed on gel electrophoresis. However, recent comparisons made between available Indica and Japonica rice genome sequences revealed that Class II SSRs were more polymorphic than Class I SSRs, but due to their small size difference polymorphism could not be achieved on agarose gel [12]. However, polymorphism can be achieved by running PCR product on polyacrylamide gel electrophoresis (PAGE) or MetaPhor® Agarose. Besides this, advancements in techniques and frequent use of capillary electrophoresis can help exploit the power of Class II SSR markers. The list of Class I and Class II markers designed in *Brachypodium,* are provided separately in the public domain (http://125.18.242.19/plantgenomedb/brachypodium_markersearch.jsp).

*Brachypodium* is a member of the Poaceae family, which comprises over 600 genera and more than 10,000 species that dominate many ecological and agricultural systems. *Brachypodium* is related to major cereal grain crops, such as wheat, barley, oat, maize, rice, rye, sorghum and millet. It has a small genome (∼272 Mbp), small physical stature, is self-fertile, has a short lifecycle and requires normal growth conditions; it also has an efficient transformation system [35]–[36]. All these qualities make it an excellent model organism for genomic research in temperate grasses and cereals. However, successful implementation of *Brachypodium* as a model plant for genomic studies in grasses requires genome-wide well-characterized molecular markers. But, only 398 SSR makers have been developed from BAC end and EST databases in *Brachypodium* [37]. The EST-derived SSR markers represent the coding region that would be important for gene tagging and cloning, however due to their low potential for polymorphism, it had marginal utility in mapping and diversity related studies [38]-[40]. In the present investigation, the large number of SSR markers developed from the genome sequence of *Brachypodium* would serve as an important genomic resource for use in many cereal crops. We have made a maiden attempt to provide a large list of SSR markers along with their primer information in the public domain.

### Database of SSR markers and genome-wide localization

To facilitate access to and utilization of SSR markers in *Brachypodium*, we developed a ***Bra*** *chypodium* **mi**crosatellite marker (**BraMi**) database, which includes details of 27,392 SSR markers. The database is hosted on the NRCPB server and is accessible at http://125.18.242.19/plantgenomedb/brachypodium_markersearch.jsp (Figure S3). The database has searchable tools to get information for all SSR markers, and useful web links of other databases, websites and research institutes related to *Brachypodium* research.

In the present investigation, we made genome-wide comparisons of SSR frequencies in monocot and dicot plants with respect to repeat motif length and sequence. Among mono-nucleotide repeats, (A/T)n was most abundant among all the plant species compared to (G/C)n. Surprisingly in the *Arabidopsis* genome, 99% of the mono-repeats were (A/T)n. Interestingly, since the CG/CG motif in the di- nucleotide repeats category contributed to less than 0.5% SSRs in dicots, it may be concluded that the CG-rich motif was least preferred in both monocot and dicot genomes. Several interesting features revealed in this study would definitely help enhance the understanding of microsatellite evolution in plants and its relationship to the divergence of monocots and dicots. In addition, the genome-wide marker resource developed in this study would be helpful for genomic studies in *Brachypodium* and other related grass species.

## Materials and Methods

### Identification of microsatellites

Genomic (pseudo molecules) and CDS sequences of *Brachypodium*, sorghum, *Populus* and *Medicago* were downloaded from the Phytozome database (www.phytozome.net/). Similarly the rice genome sequence from TIGR database (http://rice.plantbiology.msu.edu/) and *Arabidopsis* from TAIR database (www.arabidopsis.org/) were retrieved in batches. The Perl script MIcroSAtelitte (MISA) was used to identify microsatellites in all these genomes (http://pgrc.ipk-gatersleben.de/misa/). To identify the presence of SSRs, only 1 to 6 nucleotides motifs were considered, and the minimum repeat unit was defined as 10 for mono-, 6 for di-, and 5 for tri-, tetra-, penta- and hexa-nucleotides. Compound SSRs were defined as ≥2 SSRs interrupted by ≤100 bases.

### Designing SSR based primers for *Brachypodium*

The SSR information generated by MISA was used for designing primers flanking the repeats. To design primers flanking the microsatellite loci, two perl scripts were used as interface modules for the program-to-program data interchange between MISA and the primer designing software Primer3. Primer pairs were designed from the flanking sequences of SSRs using primer3_core (www.broadinstitute.org/genome_software/other/primer3.html) in batch mode via the p3_in.pl and p3_out.pl perl scripts available within the MISA package. The primer designing parameters were: 100–280 bpd amplicon size, 60°C optimal annealing temperature, 20 bp optimal primer length and 50% optimal GC content. Three sets of primer pairs were designed for each SSR to provide alternatives if amplification was unsuccessful. Redundancy of markers was checked by e-PCR by keeping a margin of 50 bp for product size; no mismatches were allowed in primer binding and the word size was kept at 11 bp [41]. The nomenclature of the primer set was B01d12 using prefix B for *Brachypodium* followed by chromosome number then d for *distachyon*, followed by SSR number as per their order from north to south on each chromosome.

All SSR markers were grouped into Class I (≥20) and Class II (12–19 bp) types. Graphical presentation of SSR distribution in different chromosomes of *Brachypodium* was made using MapChart 2.2 software (http://www.biometris.wur.nl/uk/Software/MapChart).

### Statistical analysis and functional annotation

All data were tabulated and statistical analysis was performed using SPSS 10 software package (www.spss.com). All possible SSR types were analyzed for their abundance and density per Mb for both nuclear and chloroplast genomes as well as for coding sequences. The protein sequences of genes containing GC-rich repeats were downloaded from the Phytozome database (www.phytozome.net/) and functional annotation of these genes in the *Brachypodium* genome was performed by using Gene Ontology Tools.

### Validation of SSR markers for amplification

A set of 16 *Brachypodium* genotypes was used for the validation of 55 SSR markers selected from 5 Mb intervals on all the five chromosomes (Table S5). Genomic DNA from the 16 genotypes was isolated from young leaves. The PCR reactions of 10 µl volume containing 20 ng of genomic DNA, 5 pmole each of forward and reverse primers, 0.1 mM dNTPs, 1x PCR buffer (10 mM Tris, pH 8.0, 50 mM KCl and 50 mM ammonium sulphate), 1.8 mM MgCl_2_, and 0.2 unit of *Taq* DNA polymerase was performed in a thermal cycler. The cycling conditions involved initial denaturation at 94°C for 4 min, followed by 35 cycles of denaturation at 94°C for 1 min, primer annealing at 55–60°C for 1 min, and primer extension at 72°C for 1 min. A final extension at 72°C for 7 min was done and products stored at 4°C until electrophoresis. The PCR products were resolved by electrophoresis in 3% Agarose gels in 1x TBE buffer and visualized by ethidium bromide staining.

## Supporting Information

Figure S1 Genome-wide distribution of microsatellites in monocot and dicot plant species with respect to motif length. Microsatellites were identified with criteria of mono- to hexa-nucleotides motifs using MISA software tool, and the minimum repeat unit was defined as 10 for mono-, 6 for di-, 5 for tri-, tetra-, penta-, and hexa-nucleotides.
(DOC)

Figure S2 **Distribution of SSRs motif with mono-, di-, tri-, tetra-, penta-, and hexa-repeats on the chromosome 5 of Brachypodium genome.**
(DOC)

Figure S3 **Bra**chypodium **mi**crosatellite marker (BraMi) database includes details of 27,392 SSR markers which is (a) searchable by marker Id, Location and Key words and (b) provides all the details of SSR marker including primer sequences, annealing temperature and genomic location on pseudomolecule
(DOC)

Table S1 **Distribution of microsatellite in coding DNA sequence (CDS) of monocot and dicot plant genomes.**
(DOC)

Table S2 **Functional annotation of coding GC-rich repeat containing genes in Brachypodium genome.**
(XLS)

Table S3 **Distribution of microsatellite with different motifs in chloroplast genome of six plant species.**
(DOC)

Table S4 **List of genome wide SSR markers designed for Brachypodium.**
(XLS)

Table S5 **Details of SSR markers chosen at 5 Mb intervals from the Brachypodium genome used to validate for the PCR amplification.**
(DOC)
