# Supplementary material for: Genome-Wide Distribution and Organization of Microsatellites in Plants: An Insight into Marker Development in Brachypodium
Source: PLoS One. 2011 Jun 21;6(6):e21298. doi: 10.1371/journal.pone.0021298 (PMC3119692; doi:10.1371/journal.pone.0021298)
Supplement: Figure S1 — Genome-wide distribution of microsatellites in monocot and dicot plant species with respect to motif length. Microsatellites were identified with criteria of mono- to hexa-nucleotides motifs using MISA software tool, and the minimum repeat unit was defined as 10 for mono-, 6 for di-, 5 for tri-, tetra-, penta-, and hexa-nucleotides. (DOC) [file pone.0021298.s001.doc]

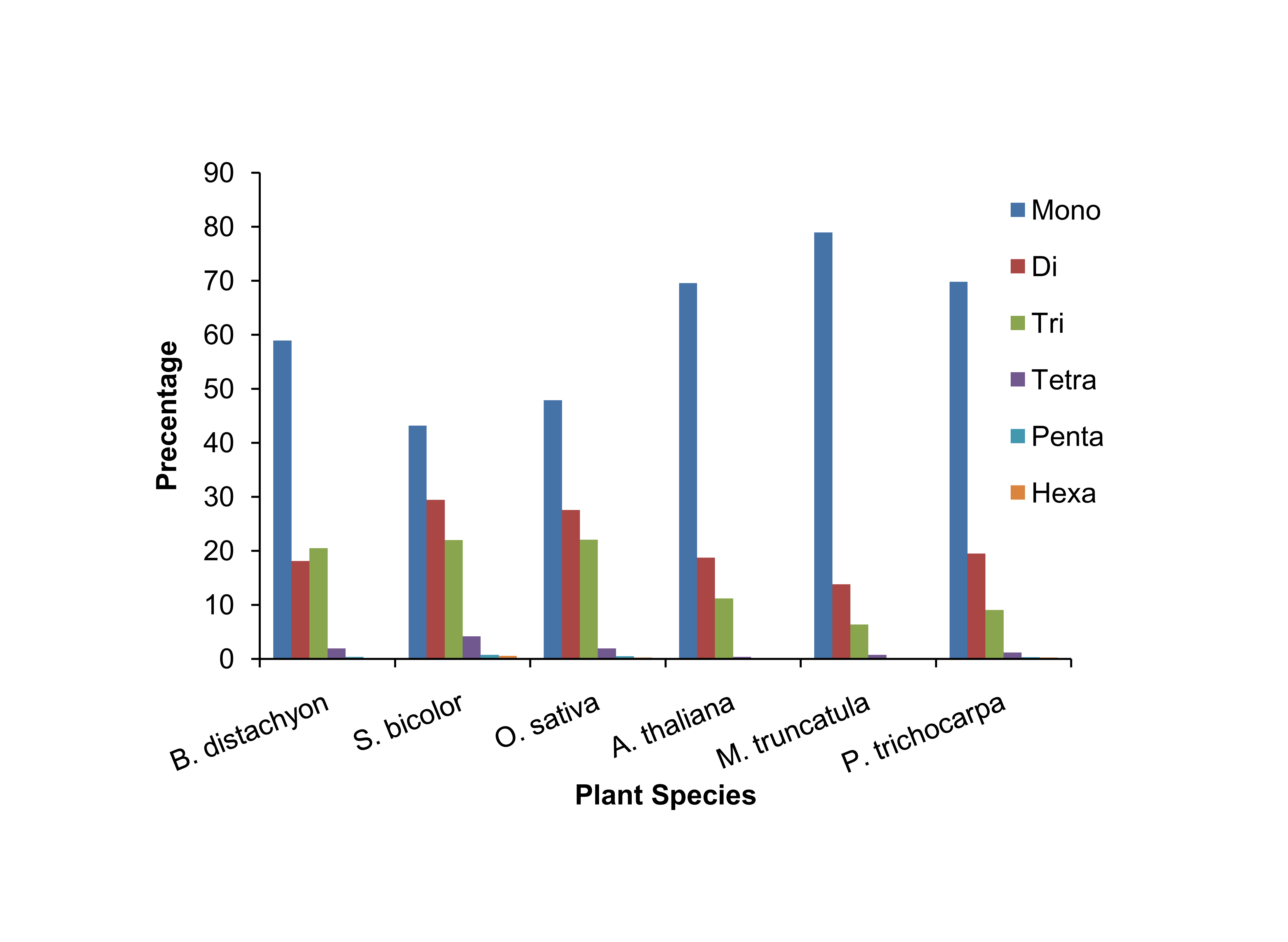


## Figure S1. Genome-wide distribution of microsatellites in monocot and dicot plant species with respect to motif length. Microsatellites were identified with criteria of mono- to hexa-nucleotides motifs using MISA software tool, and the minimum repeat unit was defined as 10 for mono-, 6 for di-, 5 for tri-, tetra-, penta-, and hexa-nucleotides.
