# Supplementary material for: Genome-Wide Distribution and Organization of Microsatellites in Plants: An Insight into Marker Development in Brachypodium
Source: PLoS One. 2011 Jun 21;6(6):e21298. doi: 10.1371/journal.pone.0021298 (PMC3119692; doi:10.1371/journal.pone.0021298)
Supplement: Figure S2 — Distribution of SSRs motif with mono-, di-, tri-, tetra-, penta-, and hexa-repeats on the chromosome 5 of Brachypodium genome. (DOC) [file pone.0021298.s002.doc]

**
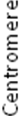
**

Mono

Di

Tri

Tetra

Penta

Hexa

**Figure S2. Distribution of SSRs motif with mono-, di-, tri-, tetra-, penta-, and hexa-repeats on the chromosome 5 of Brachypodium genome**
