# Supplementary material for: Genome-Wide Distribution and Organization of Microsatellites in Plants: An Insight into Marker Development in Brachypodium
Source: PLoS One. 2011 Jun 21;6(6):e21298. doi: 10.1371/journal.pone.0021298 (PMC3119692; doi:10.1371/journal.pone.0021298)
Supplement: Figure S3 — Brachypodium microsatellite marker (BraMi) database includes details of 27,392 SSR markers which is (a) searchable by marker Id, Location and Key words and (b) provides all the details of SSR marker including primer sequences, annealing temperature and genomic location on pseudomolecule (DOC) [file pone.0021298.s003.doc]

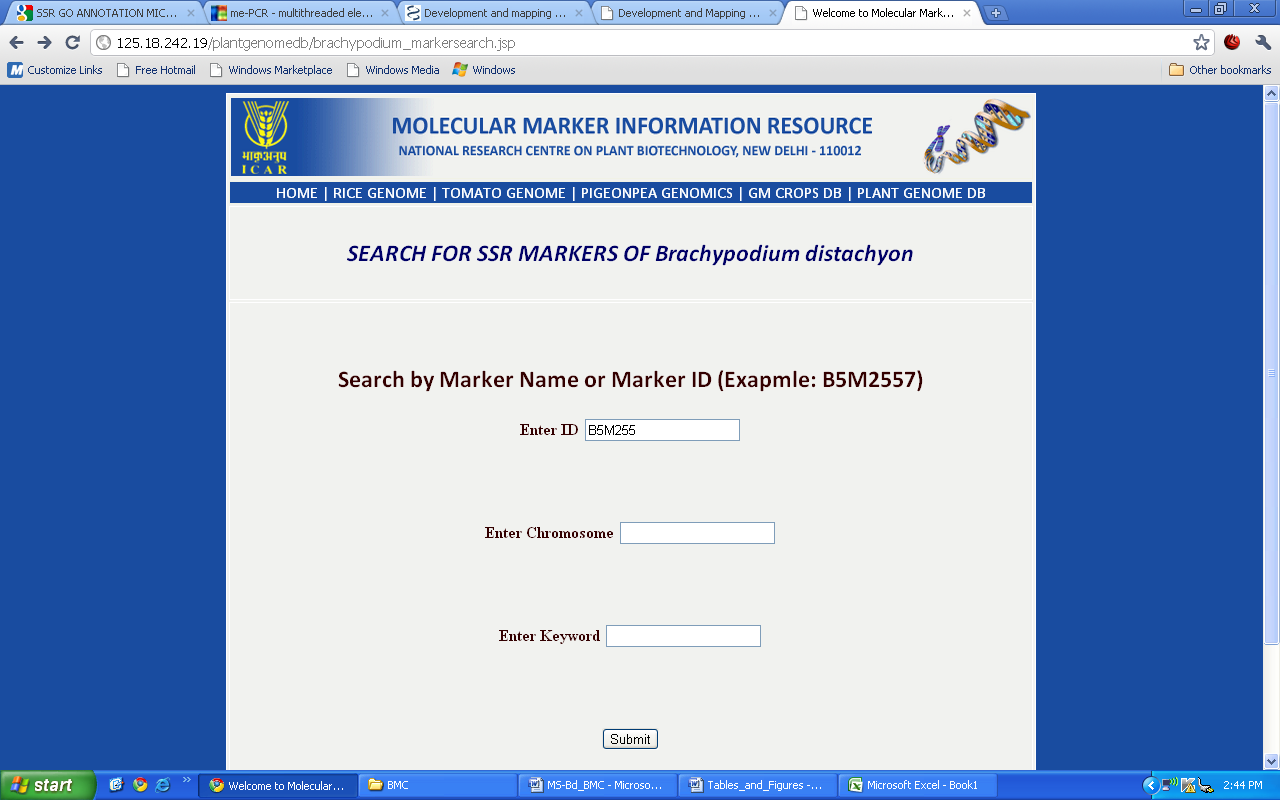


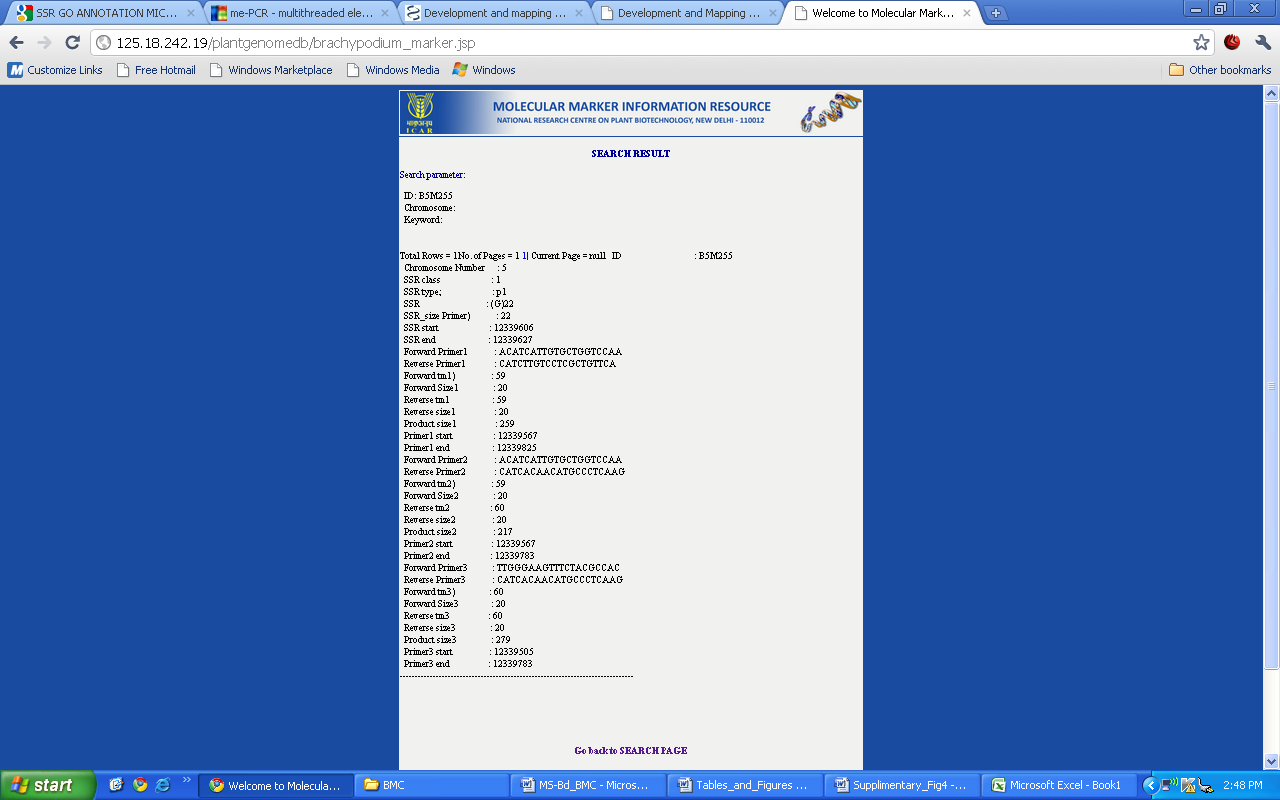


**Figure S3.** **Bra**chypodium **mi**crosatellite marker (BraMi) database includes details of 27,392 SSR markers which is (a) searchable by marker Id, Location and Key words and (b) provides all the details of SSR marker including primer sequences, annealing temperature and genomic location on pseudomolecule.
