# Supplementary material for: Genome-Wide Distribution and Organization of Microsatellites in Plants: An Insight into Marker Development in Brachypodium
Source: PLoS One. 2011 Jun 21;6(6):e21298. doi: 10.1371/journal.pone.0021298 (PMC3119692; doi:10.1371/journal.pone.0021298)
Supplement: Table S1 — Distribution of microsatellite in coding DNA sequence (CDS) of monocot and dicot plant genomes. (DOC) [file pone.0021298.s004.doc]

**Table S1. Distribution of microsatellite in coding DNA sequence (CDS) of mono**cot and dicot plant genomes

| **Characters under study** | ***B. distachyon*** | ***S. bicolor*** | ***O.***  ***sativa*** | ***A. thaliana*** | ***M. truncatula*** | ***P. trichocarpa*** |
| --- | --- | --- | --- | --- | --- | --- |
| CDS examined | 25,532 | 34,496 | 56,626 | 27,344 | 53,423 | 41,377 |
| Size of CDS (Mb) | 33.21 | 40.00 | 77.72 | 33.07 | 39.13 | 46.17 |
| CDS carrying SSR | 3,191 | 5,254 | 10,438 | 2,260 | 3,009 | 2,535 |
| Perfect SSRs | 3,796 | 6,707 | 14,387 | 2,591 | 3,384 | 2,935 |
| Compound SSR | 206 | 535 | 1,446 | 158 | 229 | 211 |
| **Total SSR** | **4,002** | **7,242** | **15,833** | **2,749** | **3,613** | **3,146** |
| **Frequency (SSRs/Mb)** | **120.5** | **181.1** | **203.7** | **83.1** | **92.3** | **68.1** |
