# Supplementary material for: Genome-Wide Distribution and Organization of Microsatellites in Plants: An Insight into Marker Development in Brachypodium
Source: PLoS One. 2011 Jun 21;6(6):e21298. doi: 10.1371/journal.pone.0021298 (PMC3119692; doi:10.1371/journal.pone.0021298)
Supplement: Table S3 — Distribution of microsatellite with different motifs in chloroplast genome of six plant species. (DOC) [file pone.0021298.s006.doc]

**Table S3. Distribution of microsatellite with different motifs in chloroplast genome of six plant species**

|  | **Bd** | **Sb** | **Os** | **At** | **Mt** | **Pt** |
| --- | --- | --- | --- | --- | --- | --- |
| A/T | 12 | 20 | 12 | 68 | 76 | 116 |
| C/G | 1 | 5 | 0 | 1 | 0 | 1 |
| AG/CT | 1 | 0 | 0 | 0 | 0 | 0 |
| AT/AT | 3 | 0 | 0 | 6 | 7 | 4 |
| AAT/ATT | 0 | 0 | 0 | 2 | 0 | 1 |
| AGT/ATC | 0 | 0 | 0 | 0 | 1 | 0 |
| **Total** | **17** | **25** | **12** | **77** | **84** | **122** |
| Size (Bp) | 135,199 | 140,754 | 134,525 | 154,478 | 124,033 | 157,033 |
| **SSR/Mb** | **125.7** | **177.6** | **89.2** | **498.4** | **677.2** | **776.9** |
