# Supplementary material for: Genome-Wide Distribution and Organization of Microsatellites in Plants: An Insight into Marker Development in Brachypodium
Source: PLoS One. 2011 Jun 21;6(6):e21298. doi: 10.1371/journal.pone.0021298 (PMC3119692; doi:10.1371/journal.pone.0021298)
Supplement: Table S5 — Details of SSR markers chosen at 5 Mb intervals from the Brachypodium genome used to validate for the PCR amplification. (DOC) [file pone.0021298.s008.doc]

**Table S5. Details of SSR markers chosen at 5 Mb intervals from the Brachypodium genome used to validate for the PCR amplification**

| **Sr. No** | **Id** | **Type** | **length** | **location** | **G/IG** | **e-PCR** | **PCR Amplification** |
| --- | --- | --- | --- | --- | --- | --- | --- |
| 1 | B1M1 | p2 | 24 | 173386 | G | no | A |
| 2 | B1M161 | c | 115 | 5020897 | IG | yes | NA |
| 3 | B1M292 | p2 | 20 | 10001067 | G | yes | NA |
| 4 | B1M456 | c | 93 | 15010088 | G | yes | NA |
| 5 | B1M603 | p2 | 20 | 20009292 | IG | yes | A |
| 6 | B1M726 | p2 | 46 | 25006179 | G | yes | A |
| 7 | B1M888 | c | 111 | 30063594 | IG | yes | NA |
| 8 | B1M1011 | p3 | 39 | 35004712 | IG | yes | NA |
| 9 | B1M1092 | p2 | 28 | 40047120 | IG | yes | A |
| 10 | B1M1210 | c | 44 | 45056545 | IG | yes | NA |
| 11 | B1M1356 | c | 150 | 50066981 | IG | yes | A |
| 12 | B1M1483 | p2 | 50 | 55083099 | IG | yes | A |
| 13 | B1M1629 | p4 | 20 | 60025296 | IG | yes | A |
| 14 | B1M1762 | c | 32 | 65010907 | IG | yes | NA |
| 15 | B1M1881 | c | 100 | 70039422 | IG | yes | NA |
| 16 | B2M1 | p3 | 21 | 1383 | G | no | A |
| 17 | B2M163 | p3 | 27 | 5030403 | G | yes | A |
| 18 | B2M285 | c* | 81 | 10032606 | IG | no | A |
| 19 | B2M439 | c | 30 | 15081649 | IG | yes | A |
| 20 | B2M569 | c* | 41 | 20027301 | IG | yes | A |
| 21 | B2M693 | p3 | 45 | 25034471 | IG | yes | NA |
| 22 | B2M792 | c | 29 | 30076588 | IG | yes | A |
| 23 | B2M899 | p3 | 24 | 35010389 | IG | yes | A |
| 24 | B2M1060 | p2 | 56 | 40002625 | IG | yes | NA |
| 25 | B2M1205 | c | 109 | 45026338 | IG | yes | A |
| 26 | B2M1322 | p2 | 26 | 50129075 | IG | yes | A |
| 27 | B2M1448 | c | 48 | 55022408 | IG | yes | A |
| 28 | B3M1 | c | 49 | 19294 | G | yes | A |
| 29 | B3M182 | p2 | 22 | 5020879 | IG | yes | A |
| 30 | B3M301 | c | 24 | 10052660 | IG | yes | A |
| 31 | B3M457 | c | 55 | 15074239 | IG | no | A |
| 32 | B3M571 | p3 | 24 | 20016458 | IG | yes | A |
| 33 | B3M659 | p2 | 20 | 25097360 | IG | yes | A |
| 34 | B3M755 | p3 | 21 | 30005585 | G | yes | A |
| 35 | B3M875 | p3 | 24 | 35067280 | IG | no | A |
| 36 | B3M998 | p1 | 25 | 40030822 | G | yes | A |
| 37 | B3M1159 | c* | 26 | 45049138 | G | yes | A |
| 38 | B3M1268 | p1 | 20 | 50002633 | IG | yes | A |
| 39 | B3M1406 | p3 | 24 | 55036331 | G | no | A |
| 40 | B4M1 | p3 | 24 | 15848 | IG | no | A |
| 41 | B4M162 | c | 156 | 5004970 | IG | yes | A |
| 42 | B4M303 | c | 50 | 10057789 | IG | no | A |
| 43 | B4M429 | p2 | 66 | 15038331 | G | yes | A |
| 44 | B4M516 | p3 | 24 | 20021013 | IG | no | A |
| 45 | B4M620 | c | 96 | 25020710 | IG | yes | A |
| 46 | B4M749 | p2 | 34 | 30051428 | IG | no | A |
| 47 | B4M922 | p2 | 22 | 35116053 | IG | yes | A |
| 48 | B4M1053 | p1 | 21 | 40005533 | G | yes | A |
| 49 | B4M1184 | p1 | 20 | 45006901 | IG | yes | A |
| 50 | B5M1 | p2 | 22 | 229641 | IG | yes | A |
| 51 | B5M111 | c | 23 | 5056359 | IG | yes | A |
| 52 | B5M195 | p1 | 26 | 10034513 | IG | no | A |
| 53 | B5M300 | p2 | 20 | 15023010 | IG | yes | A |
| 54 | B5M423 | c | 40 | 20069042 | IG | yes | A |
| 55 | B5M561 | c | 67 | 25008750 | IG | yes | NA |

P1=mono-, P2=di-, P3=tr-nucleotide repeats, c=compound SSRs, G= genic and IG= intergenic, A=amplified, NA=not amplified
